# Pulmonary vein isolation by visually guided laser balloon ablation: single-center 5-year follow-up results

**DOI:** 10.1007/s10840-023-01544-6

**Published:** 2023-04-15

**Authors:** Pieter Koopman, Thalia Bekelaar, Joris Schurmans, Thomas Phlips, Dagmara Dilling-Boer, Johan Vijgen

**Affiliations:** 1 Heart Center Hasselt, Jessa Hospital, Stadsomvaart 11, 3500 Hasselt, Belgium; 2 Limburg Clinical Research Center, Hasselt University, Agoralaan, Gebouw D, 3590 Diepenbeek, Belgium

**Keywords:** Atrial fibrillation, Ablation, Pulmonary vein isolation, Laser balloon, Long-term follow-up

## Abstract

**Background:**

Visually guided laser balloon (VGLB) ablation is a balloon-based treatment for atrial fibrillation (AF) that uses a titratable laser energy source to perform pulmonary vein isolation (PVI), allowing for real-time visualization of target tissue and ablation lesions through an endoscopic camera. Few long-term data on this technique are currently available. This report presents acute efficacy, procedural data, complication rates, and long-term AF-free survival up to 5 years post-ablation.

**Methods:**

In this single-center, retrospective, observational report, 152 patients (72.4% male, mean age 60.6 ± 9.7 years, 62.5% paroxysmal AF, 598 pulmonary veins in total) treated with the first-generation VGLB system between 2014 and 2016 were included for analysis. AF ablation consisted of PVI only.

**Results:**

Acute PVI was achieved in 98.2% of veins, with first-pass isolation in 92.5%. Procedure duration of 129 min [IQR 113–150], fluoroscopy time of 15 min [IQR 11–20], and dose area product of 5016 mGy·cm^2^ [IQR 3603–8711] were recorded. During a median follow-up of 51 months [IQR 45–57], 74.3% of patients remained free of AF (78.8% for paroxysmal and 65.3% for persistent AF, *p* = 0.108). Freedom of AF at 1, 2, 3, and 4 years follow-up was 88.2%, 82.2%, 78.9%, and 74.8%, respectively. PV reconnections were identified in only 46.9% of redo procedures. The median number of PV reconnections during redo procedures was 0 [IQR 0–2]. Anti-arrhythmic drug use was significantly reduced after ablation (*p* < 0.001). The most commonly reported complications were minor vascular complications (4.6%) and transient phrenic nerve paralysis (3.3%).

**Conclusions:**

First-generation VGLB ablation demonstrated high acute isolation rates, reasonable procedure times and low complication rates. Long-term freedom from AF was 78.8% for paroxysmal AF and 65.3% for persistent AF, performing PVI only.

**Supplementary Information:**

The online version contains supplementary material available at 10.1007/s10840-023-01544-6.

## Introduction

Atrial fibrillation (AF) is the most common arrhythmia worldwide and poses a high burden on health care facilities and costs [1]. Since AF was discovered to be predominantly triggered by ectopic activity originating in the pulmonary veins (PVs) [2], catheter ablation attempting complete electrical isolation of the PVs is a well-established alternative for anti-arrhythmic drugs (AAD) in patients with symptomatic paroxysmal or (short-standing) persistent AF [3, 4], or in patients with AF and heart failure [5]. Despite high rates of acute pulmonary vein isolation (PVI), long-term efficacy is primarily limited by PV reconnections [6]. Strategies involving more extensive substrate ablation (e.g., ablation of complex fractionated atrial electrograms or additional linear ablation) have not demonstrated superior outcomes as adjunctive approaches [7, 8].

The visually guided laser balloon (VGLB) is an ablation system designed for PVI-only ablation that allows real-time visualization of target tissue through an endoscopic camera, uses a compliant balloon to optimize tissue contact in different PV anatomies and a near-infrared laser with titratable energy, which is rotated circumferentially to create overlapping ablation lesions. Previous studies report high rates of acute PVI and durability at midterm follow-up [9–16], with success rates comparable to conventional radiofrequency ablation (RFA) and cryoballoon ablation [13, 16–20]. Reported long-term results are promising [13, 21], but available evidence is limited.

Our center has been performing PVI with the VGLB catheter since 2014, and it is now part of routine clinical practice. This paper reports our long-term clinical outcomes of 152 patients treated with the first generation VGLB catheter. We report acute efficacy, procedural data (including duration, energy use, and number of lesions), complication rates and AF free survival rates.

## Methods

### Study design

In this single-center, retrospective, observational report, 152 consecutive patients with either paroxysmal or persistent AF were treated with VGLB ablation between December 17, 2014 and September 1, 2016 at Jessa hospital (Hasselt, Belgium). This represents all cases performed with the first generation VGLB catheter. No specific anatomical imaging was performed beforehand nor were any patient-specific exclusions applied except that the patient had to be a candidate for a PVI-only AF ablation procedure. Redo procedures or patients requiring more extensive substrate ablation due to pre-existing atrial flutter or atrial tachycardia (AT) or with severely dilated left atrium (echocardiographic diameter of more than 60 mm in parasternal long axis view) were not included. Patients provided informed consent prior to the ablation procedure. The study protocol was approved by the local ethics committee (study 20.80-cardio20.17).

### Visually guided laser ablation system

Ablations were performed using the first-generation HeartLight® Endoscopic Ablation System (VGLB—CardioFocus, Marlborough, MA, USA), which consists of a 12F multi-lumen catheter with a deflectable 15F femoral sheath and a compliant balloon at its tip. The compliant balloon is filled with a solution of deuterium oxide, allowing deformation to achieve PV occlusion in various anatomies. A light source illuminates areas of balloon/tissue contact, with the laser beam aiming perpendicular to the catheter shaft covering a ~ 30° arc. A 2F fiberoptic endoscope inserted through the catheter shaft allows real-time visualization of the target tissue. Ablation is performed by laser energy, titrated between 5.5 and 12W, penetrating tissue beneath the endothelial strata. Software features allow visualization of the previous lesion location and aiming of the next energy delivery to permit delivery of overlapping and continuous lesions.

### Ablation procedure

Procedures were performed under general anesthesia with invasive arterial pressure monitoring. To avoid esophageal injury, esophageal temperature monitoring was performed (Circa S-CATH™ esophageal temperature probe, Circa Scientific Inc., Englewood, CO, USA) and energy delivery was ceased if temperatures exceeded 39.5 °C. Oral anticoagulation drugs (OAC) were not interrupted before the procedure. Vitamin K antagonists were continued with target INR between 2 and 3. For direct OACs the latest dose was administered at least 12 h before the procedure. During ablation, intravenous heparin was administered to maintain an activated clotting time between 300 and 350 s.

Two separate transseptal punctures were performed: one for introduction of the VGLB catheter and a second one for a circular mapping catheter. In all cases, real-time 3D rotational angiography was performed during both atrial and right ventricular rapid pacing. The VGLB catheter was positioned at the ostium of the target PV, and progressive inflation of the balloon was performed until optimal visual occlusion was achieved. Using visual guidance, overlapping lesions were applied. During ablation of right-sided PVs, phrenic nerve pacing was performed. When loss of capture occurred, indicating possible phrenic nerve injury, ablation was interrupted. After initial PV encircling (i.e., first-pass ablation), electrical isolation was assessed with a circular mapping catheter. When necessary, additional lesions were applied at areas of conduction breakthrough. The endpoint of electrical isolation of all PVs, defined as bidirectional conduction block, was assessed at the end of the procedure.

### Follow-up

A post-procedural blanking period of 90 days was applied. However, if an AF recurrence occurred during this blanking period and resulted in a subsequent repeat procedure, this relapse was considered a recurrence and failure of the primary endpoint. A first routine follow-up occurred at our center 3 months after hospital discharge, including a 24-h Holter recording, physical examination, 12-lead electrocardiogram and assessment of adverse events. An additional Holter recording 12 months after ablation was performed. Afterwards, follow-up was ensured by the referring cardiologist. All patients reached 1 year follow-up, and final follow-up results (range 43–66 months) were available for all but 15 patients. Due to logistical and economical reasons, follow-up could unfortunately not be performed with implantable devices, but as most patients were symptomatic for their previous AF episodes, referring cardiologists performed regular ECG (at least once yearly) or holter (at least 24 h yearly during follow-up after 1 year) recordings, and the Belgian health care system is highly organized and highly accessible, we believe follow-up was reliable. Patients that did not comply aforementioned follow-up criteria were marked as lost to follow-up. Continuation or cessation of AADs was left to the discretion of the referring physician.

### Endpoints

The primary endpoint was freedom of AF or (AT) during long-term follow-up. AF recurrence was defined as a documented AF episode either on 12-lead-electrocardiogram, Holter recording or device analysis of greater than 30 s. Secondary endpoints were assessment of adverse events, first-pass PVI rate, overall acute PVI rate and number and rate of PV reconnections in patients undergoing a repeat ablation procedure. All adverse events were reported and assessed.

### Statistical methods

Statistical analyses were conducted using SPSS Statistics 26 software (IBM, NY, USA). Normality of distribution of continuous variables was tested by Kolmogorov–Smirnov test. Normally distributed continuous variables are presented as mean ± standard deviation (SD), whereas non-normally distributed variables are reported as median [interquartile ranges (IQR)]. For categorical variables, frequency and percentage are provided. Associations between categorical variables were tested using Chi-squared test or Fisher exact test in the case of small sample sizes. Odds ratio (OR) and 95% confidence interval (CI) were calculated. For comparison of paired categorical data, McNemar’s test was used. Survival analysis was performed with Kaplan–Meier curves. To compare survival curves, log-rank tests were used. A probability value of *p* ≤ 0.05 was considered statistically significant. Multivariable logistic regression analysis was performed using a Cox proportional hazard model.

## Results

### Patient demographics

Baseline characteristics are presented in Table 1. All patients were referred for ablation due to AF symptoms refractory to AAD treatment. In total, 95 patients (62.5%) were treated for paroxysmal and 57 (37.5%) for persistent AF. The majority of patients were male (*n* = 110; 72.4%). The mean age was 60.6 ± 9.7 years (range 34–82 years).

**Table 1:** Patient characteristics

	Patients (*n* = 152) *N* (%), mean ± SD, or median [IQR]	Available values (XX/152)
Age, years	60.6 ± 9.7	152/152
Sex		152/152
Male	110 (72.4)	
Female	42 (27.6)	
Atrial fibrillation		152/152
Paroxysmal	95 (62.5)	
Persistent	57 (37.5)	
BMI, kg/m^2^	27.9 ± 4.1	151/152
Smoking	23 (15.1)	152/152
Hypertension	80 (52.6)	152/152
Diabetes	14 (9.2)	152/152
Coronary artery disease	11 (7.2)	152/152
Heart failure with reduced ejection fraction (HFrEF)	13 (8.6)	152/152
Chronic kidney disease	5 (3.3)	152/152
Sleep apnea	9 (5.9)	152/152
Symptoms (mEHRA score)		152/152
mEHRA 1	14 (9.2)	
mEHRA 2a	34 (22.4)	
mEHRA 2b	48 (31.6)	
mEHRA 3	34 (22.4)	
mEHRA 4	22 (14.5)	
CHA2DS2VASC score	1 [0–3]	152/152
Atrial dimensions in PLAX		152/152
No enlargement (≤ 34 mm)	27 (17.8)	
Mild enlargement (35–41 mm)	55 (36.2)	
Moderate enlargement (42–48 mm)	43 (28.3)	
Severe enlargement (≥ 48 mm)	27 (17.8)	
Medication		152/152
Betablockers	106 (69.7)	
Anti-arrhythmic drugs	126 (82.9)	
None	8 (5.3)	

*BMI* body mass index, *HFrEF* heart failure with reduced ejection fraction, *IQR* interquartile range, *mEHRA score* modified European Heart Rhythm Association score, *PLAX* parasternal long axis view, *SD* standard deviation

### Procedural data

A total of 598 PVs were targeted across all patients (Table 2). Anatomical variations in which a PV consisted of multiple ostia requiring separate catheter positioning and ablation, were counted as multiple PVs. Acute PV isolation was achieved in 587 PVs (98.2%), with a first-pass success in 553 PVs (92.5%). Following mapping of conduction breakthrough, additional lesions were applied in 45 PVs. Failure to achieve electrical isolation despite multiple applications occurred in 11 PVs (1.8%), two of which (same patient) were due to a console error.

**Table 2:** Procedural data

	Total PV	First-pass isolation *N* (%)	Acute isolation* N* (%)	Number of lesions (*N*) median [IQR]	Energy per lesion (watt) median [IQR]	Time per lesion (seconds) median [IQR]	Time per vein (minutes) median [IQR]
LSPV	144	129 (89.6)	141 (97.9)	30 [27–35]	10 [10–11]	20 [20–22]	15 [13–19]
LIPV	143	134 (93.7)	140 (97.9)	27 [24–31]	10 [9–10]	20 [19–20]	21 [17–25]
LCPV	7	7 (100.0)	7 (100.0)	31 [31–48]	10 [10–10]	20 [18–20]	18 [17–27]
RSPV	153	143 (93.5)	152 (99.3)	30 [27–35]	10 [9–10]	20 [19–20]	15 [13–21]
RIPV	146	136 (93.2)	142 (97.3)	29 [25–34]	9 [8–10]	20 [19–21]	16 [12–21]
RCPV	1	1 (100.0)	1 (100.0)	54	9	19	51
RMPV	4	3 (75.0)	4 (100.0)	25 [16–35]	8 [7–9]	22 [19–26]	13 [6–17]
Total	598	553 (92.5)	587 (98.2)	29 [26–34]	10 [9–10]	20 [19–20]	16 [13–23]

*IQR* interquartile range, *LCPV* left common pulmonary vein, *LIPV* left inferior pulmonary vein, *LSPV* left superior pulmonary vein, *RCPV* right common pulmonary vein, *RIPV* right inferior pulmonary vein, *RMPV* right middle pulmonary vein, *RSPV* right superior pulmonary vein

**Fig. 1:**
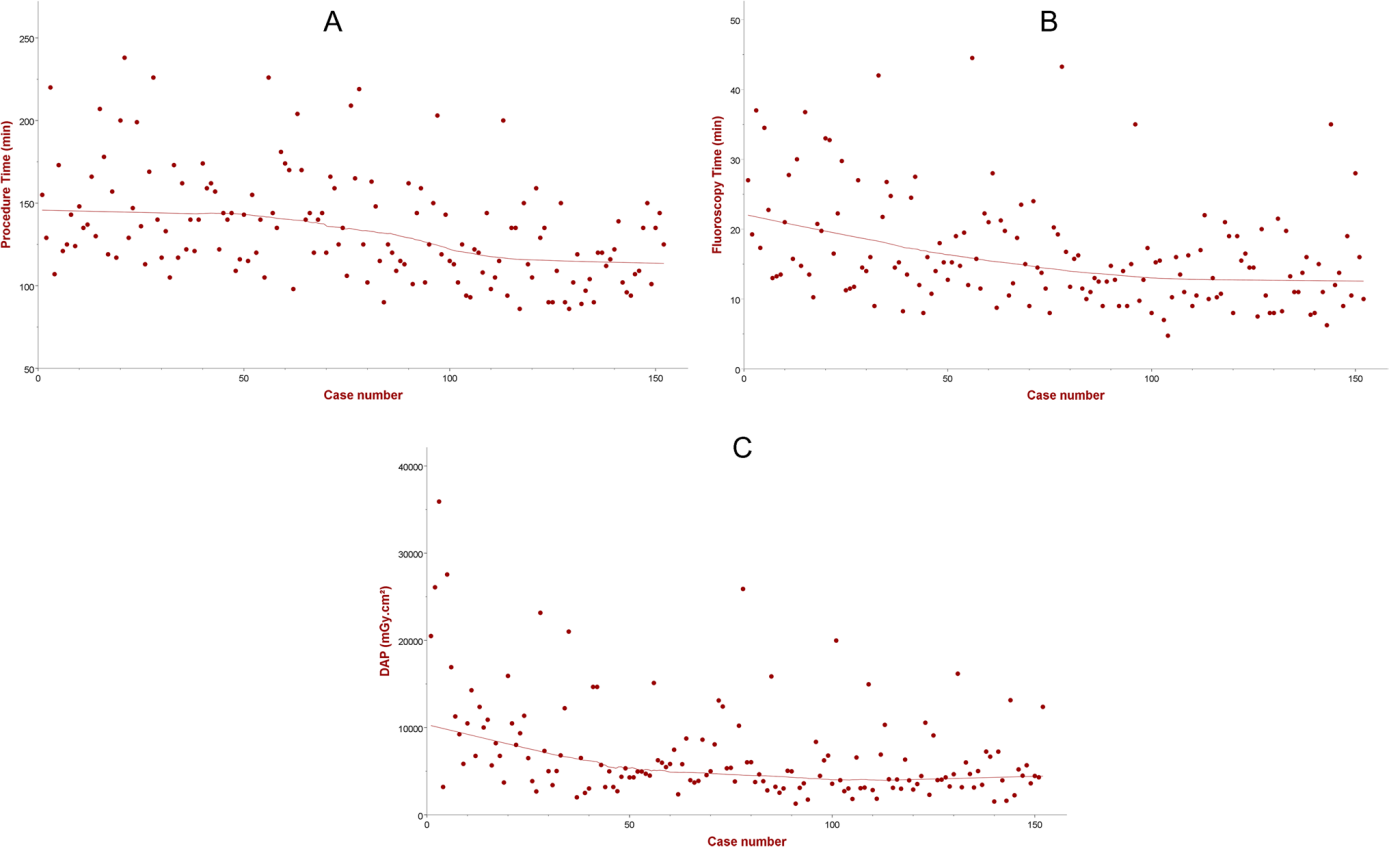
Learning curves (best fit) representing reduction in procedural duration (**A**), fluoroscopy times (**B**), and radiation dose (**C**) as experience grows

The median procedure duration (skin-to-skin) of 129 min [IQR 113–150 min] and median fluoroscopy time of 15 min [IQR 11–20 min] were recorded (Table 2). The median dose area product (DAP) delivered was 5016 mGy·cm^2^ [IQR 3603–8711 mGy·cm^2^]. There was an operator learning curve for both operators, with a significant reduction in procedure duration, fluoroscopy time, and DAP throughout the study period (Fig. 1). The procedural duration was shortened by an average of 25 min from the first ten to the last ten cases for each operator.

A median of 29 lesions [IQR 26–34] were applied in a median ablation time of 16 min [IQR 13–23] per PV (Table 2). Median energy delivery was 10 watts per lesion [IQR 9–10] during a median energy delivery time of 20 s [IQR 19–20].

### Safety and Adverse events

Peri-procedural complications were reported in 15 patients (Table 3). Minor vascular access complications were the most frequently reported complication (*n* = 7, 4.6%) and resolved without surgical intervention. Phrenic nerve paralyses (*n* = 5, 3.3%) were all transient. Two (1.3%) minor self-resolving pericardial effusions without tamponade (most likely pericarditis) and 1 (0.7%) transient ischemic attack (TIA, short-lasting diplopia the morning after ablation) occurred. No stroke, PV stenosis or atrial-esophageal fistula was observed. No severe or life-threatening complications occurred. Three patients died during follow-up due to non-procedural related causes at 6 months (suicide), 1 year (myocardial infarction) and 2 years (following abdominal surgery) after ablation.

**Table 3:** Adverse events

	*N*** (%)**
Procedural complications	
Minor vascular complications	7 (4.6)
Transient phrenic nerve paralysis	5 (3.3)
Pericardial effusion, without tamponade	2 (1.3)
Transient ischaemic attack (TIA)	1 (0.7)
Stroke	0 (0.0)
Atrial-esophageal fistula	0 (0.0)
Pulmonary vein stenosis	0 (0.0)
Death	0 (0.0)

### Clinical follow-up

**Fig. 2:**
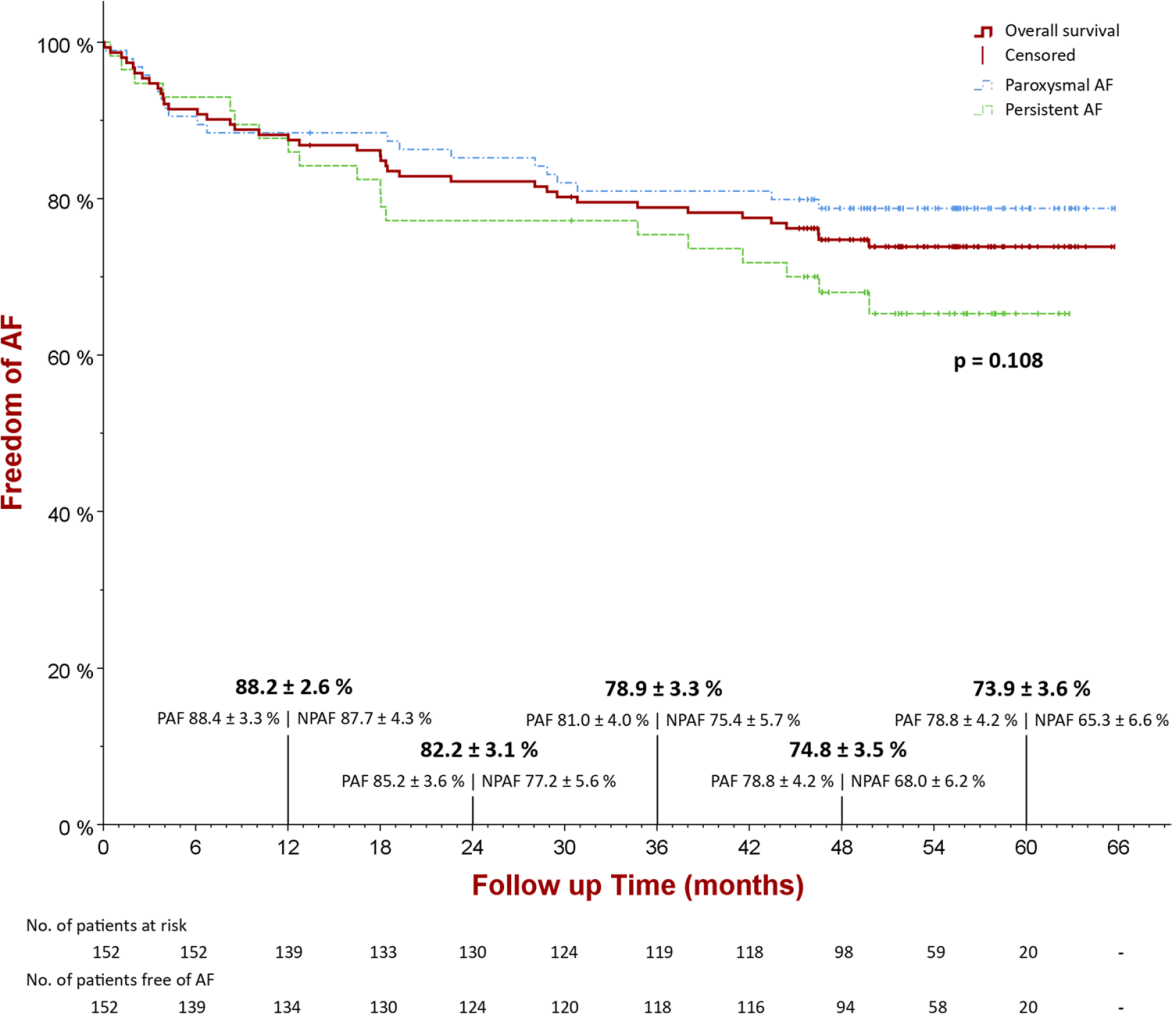
Kaplan–Meier curves of AF-free survival (on/off AAD) after visually guided laser balloon ablation in patients with non-paroxysmal (persistent) AF (green) or paroxysmal AF (blue) (p = 0.108). Estimated percentages for freedom of AF at 12, 24, 36, 48, and 60 months of follow-up are indicated

During a median follow-up of 51 months [IQR 45–57], 113 patients (74.3%) remained free of AF (78.8% for paroxysmal AF and 65.3% for persistent AF) while 39 patients had an AF recurrence. Estimated freedom from AF at 1, 2, and 4 years follow-up was 88.2%, 82.2%, and 74.8%, respectively (Fig. 2).

One hundred thirty-nine patients reached 1 year follow-up, with freedom of AF in 88.4% of paroxysmal and 87.7% of persistent AF patients. Ninety-eight patients reached 4-year follow-up, including 68 paroxysmal and 30 persistent AF patients, with freedom of AF in 78.8% of paroxysmal and 68% of persistent AF patients. Twenty patients reached 5-year follow-up, including 16 paroxysmal and 4 persistent AF patients, with freedom of AF in 78.8% of paroxysmal and 65.3% of persistent AF patients. During follow-up, there was a tendency towards more AF recurrences in persistent AF patients, although this result was not significantly different [OR = 1.88, 95%-CI 0.9–3.93, *p* = 0.108] (Fig. 2).

The presence of left atrial enlargement, defined as a left atrial diameter in the parasternal long axis of more than 40 mm, appeared to have an influence on AF recurrence, but this effect failed to reach statistical significance (Table 4). Also, additional analysis using prespecified subgroups to grade severity of atrial enlargement did not show statistical significance (*p* = 0.356). Analysis of other risk factors known to contribute to AF recurrence did not demonstrate a significant association with AF recurrence either (Table 4). In particular, first-pass isolation of all targeted PVs during ablation did not have an influence on AF recurrence (*p* = 0.578).

**Table 4:** Influence on AF recurrence, multivariable Cox proportional hazard regression model

Independent variables	*p*-value
Atrial enlargement (> 40 mm PLAX)	0.280
First pass isolation of all targeted PVs	0.578
Age	0.592
Hypertension	0.091
Obesity	0.081
Sleep apnea	0.708
Diabetes	0.516
Smoking	0.217
Coronary artery disease	0.988
Heart failure with reduced ejection fraction (HFrEF)	0.929
Chronic kidney disease	0.782

*AF* atrial fibrillation, *PLAX* parasternal long axis view, *PVs* pulmonary veins

Recurrence of AF was observed in 39 patients (25.7%), occurrence of left-sided AT in 10 patients (6.6%). Redo procedures were conducted in 32 patients (21.1%), 7 patients did not undergo a redo procedure. PV reconnections (*n* = 28) were identified in 15 patients (46.9% of redo procedures, 9.9% of total procedures). In the other patients all PVs were still isolated and the substrate causing AF was located elsewhere in the left atrium. The median number of PV reconnections was 0 [IQR 0–2]. Location of PV reconnection was variable, although most reconnections occurred in the right inferior PV and left superior PV (both *n* = 8, 28.6%), followed by the left inferior PV (*n* = 6, 21.4%), right superior PV (*n* = 5, 17.9%), and right middle PV (*n* = 1, 3.6%). In the 9 patients undergoing a redo procedure due to left-sided AT, no PV reconnection was found. AF-free survival after multiple procedures was 88.2% with an average of 1.27 procedures per patient. Choice of ablation technology for redo procedures was left to the discretion of the operator but eventually turned out to be exclusively RFA due to the anticipated limited number of PV reconnections and the possibility to ablate LA substrate.

### Medication

Before VGLB ablation, a total of 82.9% of patients received AAD. Most patients were treated with Class IC AAD (flecainide, 46.1%), or Class III AAD (amiodarone or sotalol, 38.8%), often combined with beta-blockers (69.7%). After PVI there was a significant decrease in AAD use (28.3%, *p* < 0.001) and beta-blocker treatment (59.9%, *p* = 0.032). After ablation 25.0% of patients received no AAD or beta-blockers, compared to 5.3% before ablation (*p* < 0.001). Considering only patients with sustained freedom of AF during follow-up, AAD and beta-blocker use were reduced to 23.0% (*p* < 0.001) and 60.2% (*p* = 0.016), respectively, at the time of data collection and 29.2% of patients were off all AF medications (*p* < 0.001).

## Discussion

### Findings

This experience reports real-world clinical efficacy of VGLB ablation as part of standard practice for treatment of both paroxysmal and short-standing persistent AF (< 1 year) using PVI-only ablation. Acute electrical isolation could be achieved in 98.2% of PVs. Acute procedural failure happened in 9 patients due to infrequent causes such as technical problems (console error), phrenic nerve paralysis, or inability to achieve permanent isolation of the PV due to refractory conduction in a suspected very thick myocardium. Previous cohort studies with VGLB ablation showed similar acute success rates varying between 97.7 and 100% [9–16, 21].

Following application of a first circumferential ablation lesion, 92.5% of PVs were isolated. Although in earlier studies first-pass success was lower (68 to 83%) [9, 11, 12], more recent publications report similar results (88 to 95%) [13, 14], depending on optimal tissue contact and stable catheter position [9], high-dose (> 8.5W) energy application [10], and growing experience [15]. Our high first-pass PVI success rate may be explained by our ablation approach: rotational angiography was performed in all patients ensuring optimal catheter positioning, optimal tissue contact was pursued in all PV anatomies, and high-dose energy applications (median 10 Watt) were used. These strategies also likely contributed to the long-term durability of ablation lesions. The number of lesions applied per vein was similar to those reported by other authors [9–11, 15, 17]. Since all VGLB procedures in our center were performed by only two operators, the operator’s experience advanced over a relatively short period of time, reaching optimal procedure time and radiation dose after approximately 25–50 cases each.

At 1-year follow-up, we report freedom of AF in 88.4% of paroxysmal and 87.7% of persistent AF patients. Previous reports showed 1 year freedom of AF ranging from 60 to 83% for paroxysmal AF [9–15, 18], and 70 to 75% for persistent AF [16, 17].

Available long-term results for VGLB ablation are limited. Sediva et al.[13] conducted a clinical follow-up of 192 patients with almost exclusively paroxysmal AF, reaching 75% freedom of AF after 4 years, but only 32 paroxysmal AF patients reached 4-year follow-up and none of the persistent AF patients reached 2-year follow-up. Reissmann et al.[21] conducted a follow-up of 90 patients with exclusively paroxysmal AF. Twenty-three patients reached 5-year follow-up, with a reported freedom of paroxysmal AF of 51%. In our current report, 98 patients (including 30 persistent AF patients) reached 4 years follow-up and 20 patients (including 4 persistent AF patients) reached 5-year follow-up. Estimated freedom of AF reached 78.8% for paroxysmal and 68% for persistent AF patients at 4-year follow-up and 78.8% for paroxysmal and 65.3% for persistent AF patients at 5-year follow-up. Our report equals the longest follow-up for paroxysmal AF patients with comparable results and is the first to document long-term follow-up for persistent AF patients treated with VGLB.

As expected, sustained freedom of AF was higher in patients with paroxysmal AF as compared to persistent AF, but not statistically significant (*p* = 0.108). This could be due to patient selection since VGLB was offered to persistent AF patients without extended arrhythmogenic substrate or extremely dilated atria. The numerical difference, however, was rather large, and with more power, statistical significance would possibly have been met. Subgroup analyses were performed based on the presence of certain risk factors, such as hypertension, obesity, or sleep apnea, but no statistically significant differences in the recurrence of AF were documented. Besides, the effect of these risk factors seems more convincing on the occurrence of AF in patients without previous AF ablation [22]. Although stratification by left atrial dimensions was expected to show more frequent recurrence of AF in patients with severe atrial enlargement, our results do not support this (*p* = 0.117).

Ease of use of the VGLB catheter resulted in a relatively short learning curve with an initial phase of around 15 procedures and a final phase at 25–50 procedures. Despite no previous experience with VGLB ablation, median procedure duration (129 min, IQR 113–150 min) and fluoroscopy times (15 min, IQR 11–20 min) were low and decreased progressively over time. Similar results are reported by some [9, 10, 16], although most authors report longer procedure durations and fluoroscopy times [11–14]. Studies comparing first-generation VGLB ablation to RFA [13, 16, 17] or cryoballoon ablation [18, 19] performed by experienced operators, failed to demonstrate different procedure duration.

Redo procedures were performed in 21.1% of cases. In less than half (46.9% of redo procedures, 9.9% of all procedures) PV reconnection was documented. Most redo procedures required additional substrate ablation. The median number of PV reconnections during redo procedures was 0 [IQR 0–2]. When PV reconnection was present, it manifested most frequently in the right inferior PV and left superior PV. This might be explained by difficulties obtaining optimal tissue contact at the ostium of the right inferior PV using the first-generation VGLB catheter. Second- and third-generation VGLB catheters incorporate an ultra-compliant, pear-shaped balloon with adaptable size that in our experience improves the ability to obtain contact in these areas. These second- and third-generation catheters were not used in our study cohort. Left-sided PV reconnection was often located near the appendicular ridge or anterior carina where conduction sleeves are often situated in deeper tissue layers. Right inferior and left-sided reconnections are also common with other ablation techniques, such as RFA and cryoballoon [23]. None of the patients undergoing redo ablation due to AT showed PV reconnection.

Adverse events were limited. Minor vascular complications occurred in 4.6% of patients, possibly due to new experience with a 15F femoral sheath. As a result, we no longer perform femoral invasive arterial monitoring and access points are now closed with a figure-of-8 suture instead of manual compression, largely eliminating vascular complications. Transient phrenic nerve injury occurred in 3.3% and pericardial effusion without tamponade in 1.3% of patients. These results are similar to other reports [10–16]. Phrenic nerve injury most commonly occurred when the VGLB catheter had to be pushed and advanced slightly inside the ostium of the right superior PV to obtain optimal tissue contact, thereby pushing the target tissue closer towards the phrenic nerve. Second- and third-generation VGLB catheters consisting of a more compliant balloon, allowing more proximal positioning and minimizing the need for pushing might reduce phrenic nerve injury rates. There were no reports of PV stenosis, likely due to the use of 3D rotational angiography and direct endoscopic visualization, allowing for reliable balloon positioning well outside of the PVs.

### Limitations

This retrospective report comprises a real-world experience, posing some limitations. Although routine follow-up after 3 months and 1 year was conducted at our center, further follow-up was left to the referring cardiologist. As such, long-term results are based on the patients’ latest contact with their cardiologist. This limitation is partially overcome by the low rate of patients lost to follow-up. Also notable is the post-procedural continuation of AADs in 23% of patients, due to concomitant arrhythmias (e.g., residual symptomatic atrial ectopy, but mostly ventricular ectopy or non-sustained ventricular tachycardia). Continuation of betablockers and calcium antagonists could be explained by their anti-hypertensive properties.

The presence of left atrial enlargement based on measurement of parasternal long axis dimensions only could be variable. Unfortunately, left atrial volume data were not available for many patients (*n* = 49/152) and therefore were not included in the dataset. Our report reflects a single-center experience, performing PVI only. Non-PV triggers during the index procedure were not treated. This report only contains results for the first-generation VGLB catheter. During follow-up of this cohort, the system was updated via the second and third generation VGLB systems though the energy source remained unchanged. Third-generation VGLB has been shown to significantly decrease procedure times, maintaining similar acute procedural results [24]. Long-term follow-up results for the third generation VGLB have not been published yet. To further assess clinical efficacy of this technique, larger, randomized studies are required.

Other ablation modalities are not discussed in this report. Previously published long-term results for RFA and cryoballoon PVI are slightly less optimistic [25–27]. Long-term results for promising new ablation modalities i.c. pulsed field ablation are not yet available.

## Conclusion

We investigated the long-term efficacy of VGLB ablation as part of routine clinical practice for the treatment of both paroxysmal and persistent AF. High acute isolation rates and low complication rates were documented. Long-term results were promising for both paroxysmal and persistent AF.

### Supplementary Information

Below is the link to the electronic supplementary material.

Supplementary file1 (XLSX 17 KB)

## Data Availability

The data underlying this article are available in the article and in its online supplementary material.

## Abbreviations

NPAF: Non-paroxysmal (persistent) atrial fibrillation
OAC: Oral anticoagulation drugs
PAF: Paroxysmal atrial fibrillation
RFA: Radiofrequency ablation
VGLB: Visually guided laser balloon
